# Nucleosome organizations in induced pluripotent stem cells reprogrammed from somatic cells belonging to three different germ layers

**DOI:** 10.1186/s12915-014-0109-x

**Published:** 2014-12-21

**Authors:** Yu Tao, Weisheng Zheng, Yonghua Jiang, Guitao Ding, Xinfeng Hou, Yitao Tang, Yueying Li, Shuai Gao, Gang Chang, Xiaobai Zhang, Wenqiang Liu, Xiaochen Kou, Hong Wang, Cizhong Jiang, Shaorong Gao

**Affiliations:** College of Life Science, Beijing Normal University, Beijing, 100875 China; National Institute of Biological Sciences, NIBS, Beijing, 102206 China; Clinical and Translational Research Center of Shanghai First Maternity & Infant Hospital, The School of Life Sciences and Technology, Shanghai Key Laboratory of Signaling and Disease Research, Tongji University, 1239 Siping Road, Shanghai, 200092 China; CAS Key Laboratory of Genome Sciences and Information, Beijing Institute of Genomics, Beijing, 100101 China

**Keywords:** Nucleosome organization, iPSC, ESC, Pluripotency, Chromatin remodeling, Gene expression

## Abstract

**Background:**

Nucleosome organization determines the chromatin state, which in turn controls gene expression or silencing. Nucleosome remodeling occurs during somatic cell reprogramming, but it is still unclear to what degree the re-established nucleosome organization of induced pluripotent stem cells (iPSCs) resembles embryonic stem cells (ESCs), and whether the iPSCs inherit some residual gene expression from the parental fibroblast cells.

**Results:**

We generated genome-wide nucleosome maps in mouse ESCs and in iPSCs reprogrammed from somatic cells belonging to three different germ layers using a secondary reprogramming system. Pairwise comparisons showed that the nucleosome organizations in the iPSCs, regardless of the iPSCs’ tissue of origin, were nearly identical to the ESCs, but distinct from mouse embryonic fibroblasts (MEF). There is a canonical nucleosome arrangement of -1, nucleosome depletion region, +1, +2, +3, and so on nucleosomes around the transcription start sites of active genes whereas only a nucleosome occupies silent transcriptional units. Transcription factor binding sites possessed characteristic nucleosomal architecture, such that their access was governed by the rotational and translational settings of the nucleosome. Interestingly, the tissue-specific genes were highly expressed only in the parental somatic cells of the corresponding iPS cell line before reprogramming, but had a similar expression level in all the resultant iPSCs and ESCs.

**Conclusions:**

The re-established nucleosome landscape during nuclear reprogramming provides a conserved setting for accessibility of DNA sequences in mouse pluripotent stem cells. No persistent residual expression program or nucleosome positioning of the parental somatic cells that reflected their tissue of origin was passed on to the resulting mouse iPSCs.

**Electronic supplementary material:**

The online version of this article (doi:10.1186/s12915-014-0109-x) contains supplementary material, which is available to authorized users.

## Background

Differentiated somatic cells can be reprogrammed into induced pluripotent stem cells (iPSCs) by the ectopic expression of a set of transcription factors [1]. iPSCs hold great potential for regenerative medicine without the ethical issues surrounding embryonic stem cells (ESCs). Additionally, because patient-specific iPSCs can be easily reprogrammed from differentiated somatic cells, they have a smaller risk of immunological rejection concomitant with cellular transplantation. iPSCs are similar to ESCs in a broad range of properties, such as the expression of pluripotency markers, unlimited self-renewal and the capacity to differentiate into many cell lineages, as well as the generation of viable all-iPSC mice through tetraploid complementation [2-4].

Somatic cell reprogramming involves epigenetic modification remodeling at different levels. DNA methylation is one of the well-studied epigenetic mechanisms that regulate gene expression, and it has been proposed to play an important role in reprogramming. Bisulfite genomic sequencing revealed that DNA demethylation occurred at the promoters of the pluripotency transcription factors *Oct4* and *Nanog* in the successfully reprogrammed iPSCs. Our recent study further confirmed that DNA demethylation could promote reprogramming by reactivating pluripotency genes, and we established an efficient reprogramming system by replacing *Oct4* with DNA hydroxylase *Tet1*, in conjunction with *Sox2*, *Klf4* and *c-Myc* [5].

Histone modifications are important chromatin signatures that activate or repress gene expression. For example, the methylation of histone H3 at lysines 4 and 9 are generally epigenetic marks for transcription activation and repression, respectively. Therefore, the histone modification status can greatly affect the generation of iPSCs. A recent study showed that H3K9 methylation at core pluripotency loci was a barrier to somatic cell reprogramming [6]. Comparison of the genome-wide maps of H3K4me3 and H3K27me3 occupancy demonstrated that human ESC and iPSC lines shared nearly identical profiles of these two types of histone modifications [7].

The nucleosome is the fundamental unit of eukaryotic chromatin. The characteristic nucleosomal architecture surrounding transcriptional start sites (TSSs) can influence gene regulation [8]. Densely packed nucleosomes form heterochromatin, whereas loosely packed nucleosomes constitute the relatively open euchromatin. Recent studies found that pluripotent stem cells had an open chromatin structure, and differentiated cells had a closed chromatin structure [9]. Although the aforementioned published work showed that mammalian pluripotent stem cells (ESCs and iPSCs) shared indistinguishable overall gene expression profiles, DNA methylation patterns and genome-wide maps of key histone modifications, the extent of the similarity of nucleosome positioning between iPSCs and ESCs has not yet been determined.

In our study, we established secondary induced iPSCs reprogrammed from endodermal, mesodermal or ectodermal somatic cells from full-term all-iPSC mice. We generated the genome-wide maps of the nucleosome positions using MNase-Seq, and we examined the gene expression profiles using RNA-Seq. Our results show that both the gene expression profiles and the nucleosome organization are nearly indistinguishable between iPSCs and ESCs. The subtle differences between the mouse secondary iPSC cell lines failed to reflect their tissue of origin. Active and silent genes exhibited distinct nucleosome occupancy patterns around the TSSs. Different types of transcription factor binding sites possessed characteristic topological relationships with the surrounding nucleosomes that may be important to the maintenance of pluripotency.

## Results

### Generation of secondary iPSCs from somatic cells belonging to the three different germ layers of all-iPSC mice

**Figure 1:**
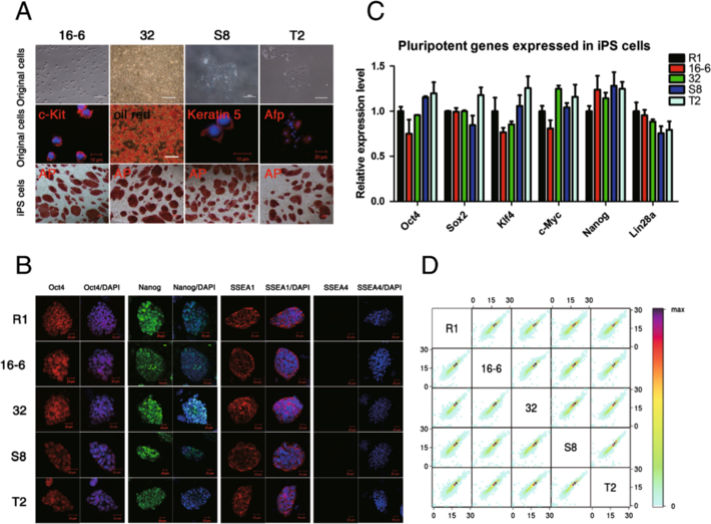
**Pluripotency and global gene expression profiles of mouse secondary iPSCs. A)** Morphology and tissue specificity of the three germ layer somatic cells before reprogramming and positive alkaline phosphatase staining after reprogramming. **B)** Immunofluorescent staining demonstrated that the R1 ESC and iPSC lines were positive for Oct4, Nanog and SSEA1 expression, but negative for SSEA4 expression. Scale bars, 20 μm. **C)** qPCR results revealed no difference in the expression levels of key pluripotency factors between the ESC R1 and the iPSC lines. **D)** The transcriptomes of the ESC R1 and four iPSC lines were highly correlated pairwise (Spearman R-values ≥0.95). The color bar shows the gene number in each comparison. The RNA-seq data were highly reproducible [see Additional file 1: Figure S1]. Secondary iPS cell lines 16-6, 32, S8 and T2 were derived from mesodermal hematopoietic cells, adipocyte progenitor cells, ectodermal epidermal cells and endodermal stomach lining cells from the all-iPSC mice, respectively. ESC, embryonic stem cells; iPSCs, induced pluripotent stem cells.

A secondary inducible iPSC system was utilized to generate iPSCs with a well-defined genetic background from three germ layer somatic cells; the similarity of the nucleosome organizations between these cells and normal ESCs was then compared. Mesodermal hematopoietic cells, adipocyte progenitor cells, ectodermal epidermal cells and endodermal stomach lining cells were collected from the all-iPSC mice, which were produced from a doxycycline-inducible iPSC line through tetraploid complementation [3,10]. The somatic cells were positive for the marker genes specific for the tissue of origin. Subsequently, the secondary iPSC lines 16-6, 32, S8 and T2 were established from mesodermal hematopoietic cells, adipocyte progenitor cells, ectodermal epidermal cells and endodermal stomach lining cells, respectively. All the iPSC lines were positive for alkaline phosphatase expression (Figure 1A). The pluripotency of the secondary iPSC lines were primarily characterized by immunocytochemical staining for pluripotency markers and by analyzing the expression of the pluripotency genes (Figure 1B and C). Moreover, the secondary iPSC lines possessed full developmental potential and produced full-term all-iPSC mice through tetraploid complementation [see Additional file 1: Figure S1A]. The efficiency of the generation of all-iPSC mice through tetraploid complementation is summarized in Additional file 2: Table S1. After the most stringent validation of pluripotency, the four iPSC lines derived from the three germ layers of the viable all-iPSC mice were selected for the following experiments.

RNA-Seq was first conducted to explore the differences in the global gene expression profiles between the secondary iPSC and the ESC lines. Our results showed high reproducibility of the genome-wide gene expression profiling [see Additional file 1: Figure S1B]. The genome-wide gene expression profiles of the secondary iPSC and the ESC lines were very similar. Additionally, the expression patterns of the secondary iPSC lines were also similar, irrespective of their tissue of origin (Figure 1D). This result is consistent with our previous studies that showed no obvious difference in gene expression patterns between mouse ESCs and four-factor and three-factor iPSCs with full pluripotency [3,11]. The gene expression profiles of dozens of human ESC and iPSC lines also shared a high degree of similarity [7]. This finding suggests that the highly similar gene expression profile forms the molecular underpinnings of stem cell pluripotency.

### Nearly indistinguishable nucleosome organization between mouse iPSCs and ESCs

**Figure 2:**
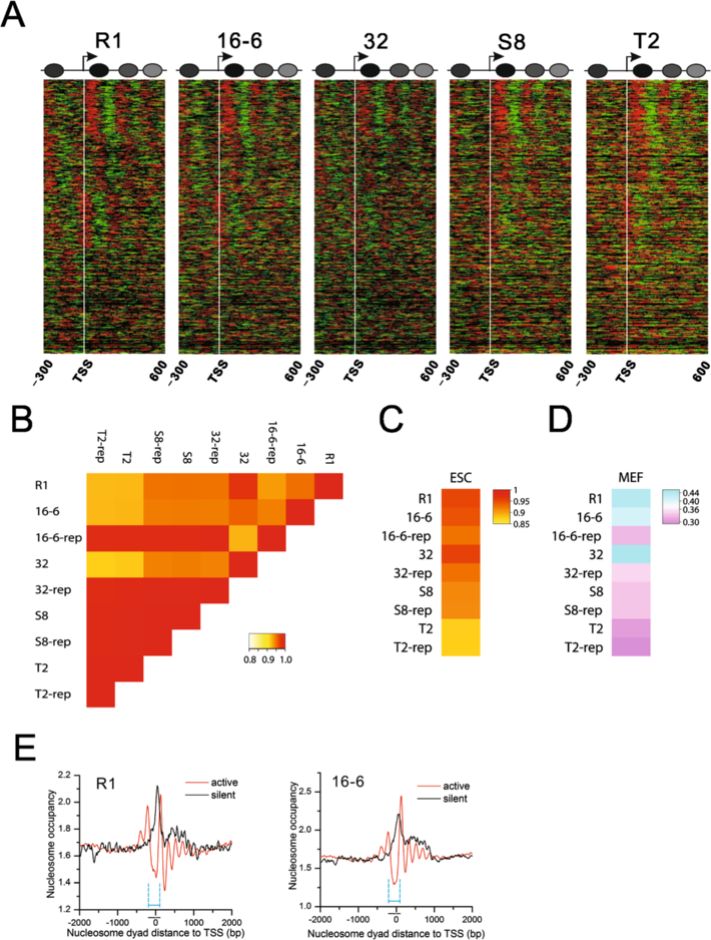
**Similar chromatin landscapes shared by mouse ESCs and iPSCs. A)** Normalized nucleosome occupancy pattern around the TSSs (indicated by white vertical line). Red represents reads (49 bp) on the sense strand. Green represents reads on the antisense strand. The adjacent red and green reads form a nucleosome. A schematic diagram that shows the canonical arrangement of -1, NDR, +1, +2, +3 nucleosomes around the TSSs was summarized from all the transcripts. **B)** We scanned the genome with a 10 kb window and calculated the normalized nucleosome occupancy for each window. Nucleosome occupancy across the genome between ESC R1 and the four iPSC lines shows an extremely high level of similarity (Spearman R-values ≥0.9). Heatmaps in Additional file 3: Figure S2B show the detailed nucleosome occupancy changes in each chromosome between the two cell lines. The high correlation between the iPS cell lines and their biological replicates indicates the high reproducibility. **C)** Global nucleosome occupancy is highly similar between the iPSCs and ESCs (Spearman R-values >0.8). **D)** Global nucleosome occupancy is significantly different between the stem cells and MEFs (Spearman R-values ≤0.4). **E)** Canonical nucleosome organization around the TSS is required for gene activation. Genes with zero read count were defined as silent genes, whereas the rest were considered active genes. There is a -1, NDR, +1, +2, +3 and so on canonical nucleosome arrangement around the TSSs of active genes. In contrast, the TSSs of silent genes were protected by one prominent nucleosome. The cyan lines indicate the region (-200 bp to approximately +100 bp of TSS) with distinct nucleosome distribution patterns between active and silent genes that are conserved in all the pluripotent stem cell lines. ESCs, embryonic stem cells; iPSCs, induced pluripotent stem cells; MEFs, mouse embryonic fibroblasts; NDR, nucleosome depletion region; TSSs, transcriptional start sites.

Genomic DNA is packaged as nucleosomes, which help regulate gene expression by controlling the accessibility of DNA. Therefore, nucleosome organization is closely linked to the physiological features and functions of cells. Several studies have shown that the chromatin of ESCs is globally open when compared with the chromatin of differentiated cells [12-14]. However, the nucleosome organization in iPSCs remains elusive. Because nucleosome positioning around the TSS plays a critical role in the regulation of gene expression [8], we first investigated the chromatin structure around the TSSs in mouse iPSCs and ESCs. We observed a canonical nucleosome arrangement of -1, nucleosome depletion region (NDR), +1, +2, +3 and so on around the TSSs in all cell lines. A close gene-by-gene examination of the nucleosome distribution around the TSS revealed no obvious differences between the iPSC and ESC lines (Figure 2A). Pairwise comparisons between the cell lines further indicated similar nucleosome organizations around the TSSs [see Additional file 3: Figure S2A]. The results were reproduced in the biological replicates [see Additional file 3: Figure S2B].

Coding regions only account for approximately 3% of the genome. To test whether the entire genome has a similar nucleosome organization between the stem cell lines, we used a 10-kb window to scan the genome. The nucleosomal read count was calculated for each window and normalized to the total number of uniquely mapped reads in each cell line. These normalized read counts were defined as the nucleosome occupancy in each window. Then, we performed a pairwise correlation analysis of the normalized nucleosome occupancy between the cell lines. The results showed an extremely high correlation. All correlation coefficients were greater than 0.9 (Figure 2B). We tried different offsets to start the scanning and obtained the same results. However, we observed a few small regions with more than two-fold nucleosome occupancy changes that were dispersed across the genome [see Additional file 3: Figure S2C]. These regions were not associated with any obvious theme. We further compared the global nucleosome occupancy between our pluripotent cells and the ESCs in the published literature [15]. The result showed very high correlation between all the cell lines indicating highly similar nucleosome organizations (Figure 2C). In contrast, the global nucleosome occupancy was significantly different between all the stem cell lines and mouse embryonic fibroblasts (MEFs) (Figure 2D). The MEF nucleosome data were mined from a previously published study [16]. All the correlation coefficients of genome-wide nucleosome occupancy between all the cell lines in this study are given in Additional file 4: Table S2.This suggests that nucleosome remodeling occurs during nuclear reprogramming and leads to a chromatin architecture similar to ESCs but different from MEFs.

### Nucleosome arrangement around the TSS is linked to gene activity

Previous studies have shown that nucleosome positioning around TSSs is uniform in yeast and *Drosophila* [17,18]. A study in human CD4^+^ T cells found more than five well-positioned nucleosomes around the TSSs of expressed genes but only one well-positioned nucleosome on the TSSs of unexpressed genes [19]. A more recent study reported that highly expressed genes had broader and more pronounced NDRs around their TSSs than lowly expressed genes in mouse ESCs and MEFs [16]. To determine the relationship between nucleosome positioning around the TSS and gene activity, we defined genes with read count equal to zero as silent genes and the rest of the genes as active genes. There was an array of well-positioned nucleosomes (from -1 to +4) around the TSSs of active genes. These TSSs are exposed to NDRs and open to transcription factors (Figure 2E and see Additional file 3: Figure S2D). In contrast, there was only one well-positioned nucleosome on the TSSs of silent genes. This nucleosome replaced the NDR and made the TSSs of silent genes inaccessible to regulators. The results suggested an open chromatin structure (NDR) on the TSS of active genes for all the pluripotent cell lines. In contrast, access to the TSS of unexpressed genes was impeded by nucleosome occupancy.

We further quantified the similarity of the nucleosome distribution pattern in the ± 2 kb region of the TSSs of active and unexpressed genes in all mouse iPSC and ESC lines. We used a 250-bp window to scan these regions of TSS and calculated the normalized nucleosome occupancy for each window. The pairwise correlation analysis of the nucleosome occupancy also showed high correlation for active and unexpressed genes between all cell lines, respectively. The Pearson correlation coefficient values of pairwise comparisons are 0.50 to 0.94 for active genes and unexpressed genes, respectively [see Additional file 5: Table S3]. We obtained the same results when using different scan window sizes. Note that the correlation coefficients between S8/T2 and other cell lines are not as high as the coefficients between R1, 16-6 and 32 cell lines. This is largely due to the variance in nucleosome distribution in the 500 to 1,000 bp downstream of the TSS (Figure 2E and Additional file 3: Figure S2D). Overall, the nucleosome distribution pattern in the region flanking the TSS is highly similar between all pluripotent cell lines and is associated with gene activity.

### Different classes of transcription factor binding sites possess characteristic topological relationships with nucleosomes

**Figure 3:**
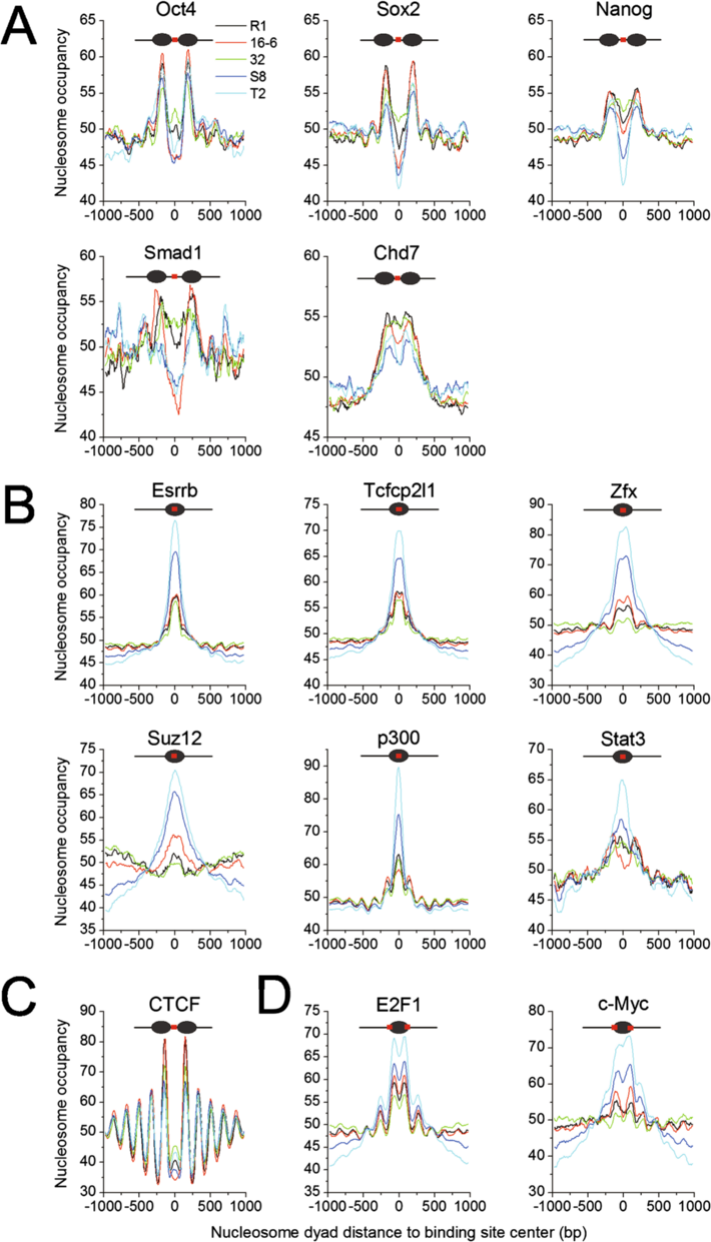
**Nucleosome occupancy at the binding sites of pluripotency-related transcription factors, protein regulators and enzymes. A)** The binding sites of the pluripotency transcription factors (Oct4, Sox2 and Nanog), the pluripotency regulator Smad1 in the BMB signaling pathway and the chromatin remodeler Chd7 were nucleosome depleted. **B)** The binding sites of self-renewal regulators (Esrrb, Tcfcp2l1 and Zfx), the PRC2 component Suz12, the coactivator p300 and Stat3 in the LIF signaling pathway were enriched on nucleosomes. **C)** The binding sites of the insulator-binding protein CTCF resided in the linker region with nucleosomal periodicity. **D)** The binding sites of factors E2F1 and c-Myc were enriched at the borders of the nucleosomes.

Pluripotency is established through complex gene regulatory networks composed of transcription factors and other regulators. Thus, the accessibility of their DNA binding sites is important in maintaining pluripotency. To gain insights into the nucleosome occupancy at these sites, we examined the binding sites that were experimentally determined using chromatin immunoprecipitation (ChIP)-seq in ESCs, including a dozen important pluripotency factors [20], the *p300* histone acetyltransferase [21] and the chromatin remodeler *Chd7* [22]. Then, the nucleosome occupancy surrounding the binding sites was calculated. Four types of nucleosome occupancy patterns were observed at the binding sites (Figure 3 and Additional file 6: Figure S3). (1) The binding sites of the core pluripotency factors (*Oct4*, *Sox2* and *Nanog*), the regulator Smad1 and the chromatin remodeler *Chd7* preferentially resided in the linker regions between two adjacent nucleosomes (Figure 3A and Additional file 6: Figure S3A). *Smad1* is the key component of the BMP signaling pathway that is linked to the core pluripotency network. Chromatin remodeling factors provide a means for crosstalk by occupying the target genes of the core pluripotency factors [23]. This setting with nucleosome depletion may allow the core pluripotency factors to bind to their target genes relatively easily and construct the core pluripotency networks during reprogramming. Intriguingly, it was reported that these core pluripotency factors also functioned as ‘pioneer factors’ and bound to closed chromatin at the first 48 hours of reprogramming [24]. The inconsistence between this finding and our own is probably due to the different time point of binding. The reported initial binding of the ‘pioneer factors’ was done at 48 hours after reprogramming induction whereas the ChIP-seq data used in our study was from ESCs that correspond to mature iPSCs. Their binding sites are not entirely the same at the two stages. Indeed, the ‘pioneer factors’ predominantly bound distal to the TSS at 48 hours post-induction but close to the TSS in ESCs [24]. Therefore, to reveal how the initial binding of the ‘pioneer factors’ changes nucleosome organizations during reprogramming requires stepwise maps of nucleosome position and binding of the ‘pioneer factors’. Additionally, the different binding patterns of the ‘pioneer factors’ at different stages may have distinct roles in reprogramming. The initial binding of the ‘pioneer factors’ promoted apoptosis and senescence and removed cells with aberrant transcription [24]. The binding pattern in ESCs or mature iPSCs in our study may play important roles in pluripotency maintenance. (2) In contrast, the binding sites of the self-renewal regulators (*Essrb*, *Zfx* and *Tcfcp2l1*), *Suz12*, which is a core subunit of *PRC2* complex, the *p300* histone acetyltransferase, and the transcription factor Stat3 were enriched on nucleosomal DNA (Figure 3B and Additional file 6: Figure S3B). Leukemia inhibitory factor (*LIF*) activates Stat3 during the maintenance of the self-renewing state [20]. The accessibility of the gene regulatory elements on nucleosomal DNA is regulated through its rotational setting [17]. Therefore, these factors may bind to their target sites following nucleosome rotation. (3) Intriguingly, *CTCF* prefers to bind the linker regions with a regular periodicity based on the nucleosome size (Figure 3C and Additional file 6: Figure S3C). This result is consistent with the nucleosome arrangement around the *CTCF*-binding sites in CD4^+^ T cells [25]. This unique setting is beneficial to *CTCF’s* role as the insulator binding protein and helps to demarcate the repressed and active chromatin regions. Notably, a small nucleosome occupancy peak at the binding sites indicated that a tiny fraction of binding sites resided on nucleosomes. (4) The last type of pattern showed binding sites on nucleosomal DNA but with an enrichment at the borders of the nucleosomes. The binding sites of the cell-cycle regulator *E2F1* and the pluripotency factor c-Myc belong to this category (Figure 3D and Additional file 6: Figure S3D). It has been reported that the *c-Myc* network is separate from the core pluripotency network [23]. Therefore, it is no wonder that the binding sites of *c-Myc* have a different topological relationship with nucleosomes than those of *Oct4*, *Sox2* and *Nanog*. Taken together, the members of the pluripotency network re-establish and maintain pluripotency during reprogramming by binding to their target sites with characteristic spatial distribution on nucleosomes.

### No impression of the tissue of origin was observed in mouse secondary iPSCs

**Figure 4:**
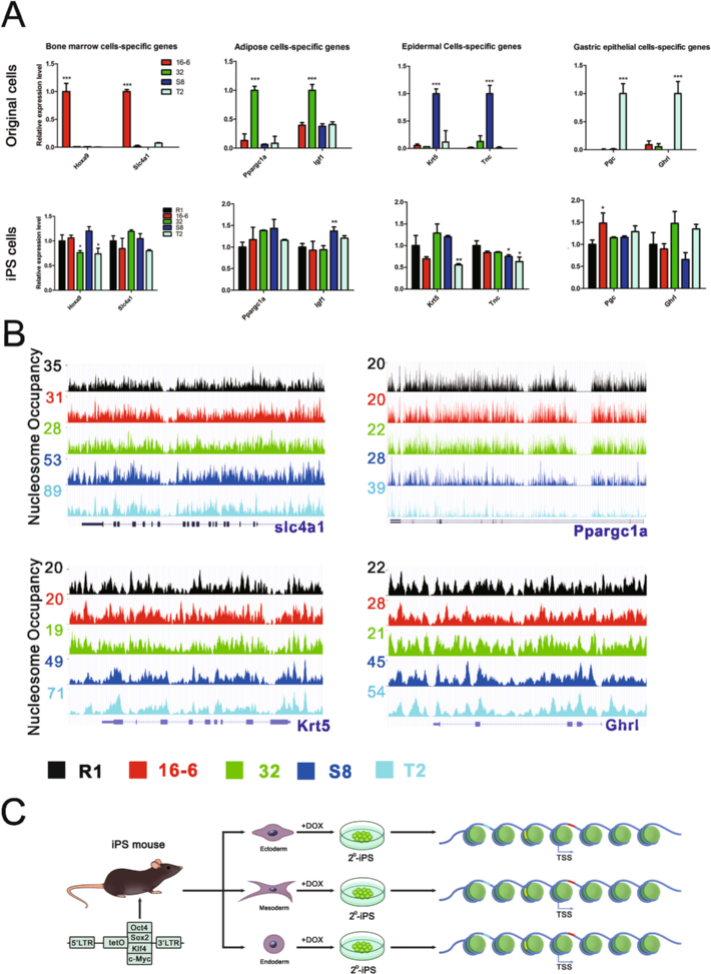
**Accurate nucleosome remodeling during nuclear reprogramming. A)** qPCR results of the germ layer specific genes showed that their expression is specific in the tissue of origin before reprogramming (top panel), but their expression in the pluripotent cells fails to reflect the tissue of origin of iPSCs (bottom panel). **B)** Nucleosome occupancy in the four representative genes in A is similar between the ESC R1 and iPSC cell lines. Transcripts from RefSeq are shown under the tracks. More loci are given in Additional file 6: Figure S3. **C)** A schematic diagram demonstrates the generation of the mouse secondary iPSCs and their nearly indistinguishable nucleosome organizations, irrespective of their tissue of origin. The transcription factor binding sites (short colored lines) were distributed across the genome in a manner consistent with the characteristic topological relationships with nucleosomes, and this distribution was similar between the iPSC cell lines. ESC, embryonic stem cell; iPSCs, induced pluripotent stem cells.

A recent study comparing the gene expression profiles of human ESCs and iPSCs observed that the persistent residual gene expression inherited from the parental fibroblast cell line could reflect their tissue of origin [26]. This raised the question of whether this is the case for the mouse secondary iPSCs. To address this question, we chose representative featured genomic loci, including pluripotency genes and tissue specific genes that bear the ‘footprint’ of their tissue of origin. The qPCR results showed that there was no difference in the expression level of the pluripotency genes (*Oct4*, *Sox2*, *Klf4*, *c-Myc*, *Nanog* and *Lin28*) between ESCs and iPSCs (Figure 1C). The nucleosome occupancy at these gene bodies was also highly similar between all the stem cell lines [see Additional file 7: Figure S4]. Interestingly, the bone marrow cell-specific gene *Slc4a1* was highly expressed only in the original somatic cells of the iPS 16-6 cell line before reprogramming. However, there was no difference in its expression level or nucleosome occupancy between the ESC R1 cells and the iPSCs from all three germ layers (Figure 4A and B). We observed the same results for the other examined adipose cell-, epidermal cell-, and stomach lining cell-specific genes. Our results suggest that no parental expression program or nucleosome organizations were left in the resulting iPSCs. Interestingly, a systematic inspection of H3K4me3 and H3K27me3 signaling in dozens of human iPSC and ESC lines revealed negligible differences between iPSCs and ESCs, and these small differences did not reflect their cell of origin [7]. As a matter of fact, the residual gene expression program of their cell of origin obtained in human iPSCs was likely due to the incomplete reprogramming [26]. Unlike human iPSCs, the four murine secondary iPSCs in our study shared the same genetic background and passed the most stringent validation of pluripotency through tetraploid complementation. Therefore, the reprogramming process accurately re-established nucleosome organizations highly similar to ESCs. The reconstructed nucleosome landscape in turn provided a chromatin setting of accessibility of DNA sequences (for example, transcription factor binding sites) that remarkably resembled ESCs (Figure 4C). As a consequence, the gene expression profiles are highly similar between ESCs and the secondary iPSCs, irrespective of their tissue of origin. No residual expression program or nucleosome positioning of the parental somatic cells that reflected their tissue of origin were passed onto the resulting iPSCs.

Notably, some iPSC’s parental tissue-specific genes were significantly differentially expressed in the iPSCs derived from other tissue cells rather than the parental tissue cells. For example, the adipose cell-specific gene *Igf1* was significantly highly expressed in the iPSC line S8 derived from epidermal cells instead of in the iPSC line 32 derived from adipocyte progenitor cells. In contrast, the expression level of *Igf1* was similar between ESC line R1, the iPSC line 32 derived from adipocyte progenitor cells, and the iPSC lines 16-6 and T2 derived from other tissues. Similar results were observed for the tissue-specific genes *Hoxa9*, *Krt5*, *Tnc* and *Pgc* (Figure 4A and B). These inconsistent differences in expression of some iPSC’s parental tissue-specific genes between the iPSCs and ESCs failed to reflect their tissue of origin. Moreover, the differences implied that the iPSCs were not identical to one another and not identical to ESCs, either.

The somatic cell reprogramming also involves re-establishment of epigenetic marks. It has been reported that the level of DNA methylation at the promoters of the pluripotency transcription factors in iPSCs was restored to as low as in ESCs [1,5,11]. However, whether the global epigenetic signatures (DNA methylation and histone modifications) are highly similar between murine iPSCs and ESCs and there are no residual parental epigenetic signatures passed to iPSCs, requires genome-wide comparisons.

## Discussion

Mammalian iPSCs possess the key characteristics of pluripotency, particularly full developmental potential through tetraploid complementation, and have given great promise to regenerative medicine. Thus, it is critical to comprehensively compare iPSCs with ESCs before the clinical application of iPSCs. Mouse secondary iPSCs generated in our lab are an extraordinary model to inspect the degree of difference between iPSCs and ESCs because secondary iPSCs meet the ESC gold-standard for the full-term development of germ-line transmittable all-iPSC mice. Moreover, secondary iPSCs have a genuine identical genetic background, in terms of the same genome and the same integration sites of the exogenous pluripotency factors. We performed a detailed inspection of the gene expression profile and chromatin structure of mouse ESC and secondary iPSCs derived from different tissues of all-iPSC mice. The overall transcriptional profiles and nucleosome occupancy were both remarkably similar between ESCs and iPSCs. Nucleosome arrangement influences gene expression, and an array of well-positioned nucleosomes were found located around the TSSs of active genes, with a NDR upstream of the TSSs. Contrary to this, only one phased nucleosome resided at the TSSs of silent genes, blocking access to these TSSs. Interestingly, transcription factor binding sites show characteristic topological relationships with nucleosomes, which contribute to the re-establishment and maintenance of pluripotency.

It is a longstanding controversy whether ESCs and iPSCs belong to a bona fide identical cell type. A large body of studies has shown consistent differences between human ESCs and iPSCs at the level of gene expression [26-29], protein expression [30], DNA methylation [28,29,31] and histone marks H3K9me3 and H3K27me3 [32]. Moreover, there is an accumulating body of evidence implying genetic differences (copy number variation and point mutation) between human ESCs and iPSCs [33-35]. All these findings suggest that human iPSCs are a unique subtype of pluripotent cell. Conversely, a study comparing the gene expression profiles of ESCs, iPSCs and fibroblasts on a large scale (dozens of cell lines) revealed no consistent differences to distinguish human iPSCs from ESCs. Further comparison of the genome-wide maps of H3K4me3 and H3K27me3 showed no significant difference in either of the histone modifications between ESCs and iPSCs. Their results supported that human ESCs and iPSCs were nearly identical cell types. Additionally, the differences in gene expression between human ESCs and iPSCs were more likely attributed to laboratory-specific biases, such as cell culture conditions, RNA extraction methods and so on. [7]. It is noteworthy to point out that it is a challenge to compare gene expression profiles and epigenetic modifications of iPSCs and ESCs because of differences in the homogeneity of cell populations and data processing, especially in the comparative analysis of data from different laboratories and across platforms. In addition to this, most of the iPSCs used in the previous studies are not genuinely genetically identical because the integration sites of the exogenous transcription factors in the genome are not the same. As a consequence, all kinds of noise in the data largely weaken the comparison conclusion.

Murine reprogramming is a similar process to human reprogramming. A prominent advantage of murine reprogramming is quality estimation by tetraploid complementation assay. In our study, the four secondary iPSC lines derived from three germ layers all passed the most stringent validation of pluripotency through tetraploid complementation and produced viable all-iPSC mice. Moreover, secondary iPSC lines should have the same genetic background. All these together make the iPSCs remarkably resemble ESCs and lead to surprisingly similar gene expression profiles (Figure 1), nucleosome organizations (Figure 2), and characteristic nucleosomal architecture of transcription factor binding sites (Figure 3) between iPSCs and ESCs. However, there still exist differences in gene expression profiles and nucleosome landscape between murine iPSCs and ESCs. These differences may possibly exert subtle effects on differentiation, and particularly influence their tumorigenicity. Moreover, integration of exogenous pluripotent transcription factors into the genome may also cause genetic variations in iPSCs. Therefore, it is hard to tell whether the murine secondary iPSCs and ESCs are a bona fide identical cell type on the basis of our results alone. Instead, it is likely that the murine secondary iPSCs are also a unique subtype of pluripotent cell by referring to the accumulating body of evidence in human reprogramming studies. The inconsistent differences in expression of some iPSC’s parental tissue-specific genes between the iPSCs and ESCs may also support this view (Figure 4A and B).

Unlike a previous study in human iPSCs [26], both gene expression profiling and nucleosome organization analysis in our study did not find impression of the tissue of iPSCs’ parental fibroblast cells. The map of mononucleosome positions in our study had high resolution with a single nucleosome. Histone modifications are nucleosomes with chemically modified histone tails. Therefore, nucleosome maps by MNase-seq or ChIP-seq have higher or equal resolution compared to histone modification maps. The previous study compared the genome-wide maps of H3K4me3 and H3K27me3 and did not find epigenetic signatures that could reflect the origin of human iPSCs [7]. Thus, no impression of tissue of origin in our study is not likely due to the lack of the resolution of the nucleosome position map. As a matter of fact, the previous study that observed impression of tissue of origin in the resulting human iPSCs used data from different tissues in different laboratories [26]. Their observation of tissue of origin was likely attributed to laboratory-specific biases, including cell culture conditions, data processing methods, and so on. [7].

Chromatin is reorganized during reprogramming. The re-established nucleosome organizations in iPSCs that can produce full-term all-iPSC mice through tetraploid complementation (referred to as ‘fully-pluripotent’ iPSCs) are highly similar to ESCs, but significantly different from MEFs in our study. However, it is unclear to what degree the nucleosome organizations of the ‘fully-pluripotent’ iPSCs resemble the ‘non-fully-pluripotent’ iPSCs. Actually, it is a challenge to address this issue because the ‘non-fully-pluripotent’ iPSCs are a wide variety of iPSCs including iPSCs only capable of differentiation into the three germ layers, formation of teratomas, embryoid body, chimeric mice or dead all-iPSC pups, respectively. Beside nucleosome organizations, there should be various mechanisms leading to the incomplete reprograming in ‘non-fully-pluripotent’ iPSCs. It will be very difficult to have a subtype of iPSCs that can represent the category of ‘non-fully-pluripotent’ iPSCs. Taken together, we do not know whether there exists a nucleosome organization pattern that could distinguish the ‘fully-pluripotent’ iPSCs from ‘non-fully-pluripotent’ iPSCs at this point. Notably, all the aforementioned studies, including our study, focused on the start and end stages of reprogramming. However, both reprogramming and nucleosome remodeling are dynamic processes. An epigenetic roadmap depicting the detailed time when, and genomic loci where, nucleosomes are disassembled and reassembled during reprogramming will reveal its molecular mechanisms.

## Conclusions

Our results reveal that both gene expression profiles and nucleosome organizations are remarkably similar between mouse ESCs and the secondary iPSCs derived from the three germ layers. They suggest that the somatic cell reprogramming process can restore the nucleosome landscape that highly resembles ESCs. Consequently, the chromatin remodeling provides a similar setting for accessibility of DNA sequences that gives rise to characteristic topology relationships of transcription factor binding sites with nucleosomes and nucleosome distribution patterns in active and silenced genes in iPSCs highly similar to ESCs. Our study helps in understanding the re-establishment of nucleosome arrangement during nuclear reprogramming and highlights its roles in the regulation of accessibility of DNA sequences and gene expression.

## Methods

### Cell culture

Mesodermal adipocyte progenitor cells, hematopoietic progenitor cells, endodermal gastric epithelial cells, and ectodermal epidermal cells were collected from eight-week-old all-iPSC mice. Briefly, adipocyte progenitor cells were derived from the stoma-vascular fraction (SVF) of the inguinal fat depots as previously described [36]. Hematopoietic progenitor cells were derived from bone marrow using c-Kit (CD117) magnetic beads (Miltenyi Biotec, Bergisch Gladbach, Germany), and were temporarily stored in Iscove's modified Dulbecco's medium (IMDM) medium containing 2% heat-inactivated fetal bovine serum (FBS), and then were induced with doxycycline (DOX). Glandular stomach mucosa was scraped and digested with 0.1% collagenase I for 40 minutes at 37°C, then cultured in basal chemically defined medium [37]. Epidermal cells were derived from abdominal skin as previously described [38].

To induce the generation of secondary iPSCs, the culture medium was replaced with embryonic stem (cell) (ES) medium supplemented with 1 μg/ml DOX; the expression of the four reprogramming factors (*Oct4*, *Sox2*, *c-Myc* and *Klf4*) in these somatic cells can be induced by the addition of DOX to the culture. ES-like colonies appeared at approximately seven to ten days, and four days after the withdrawal of DOX, the smooth colonies were picked up and passaged with trypsin every one to three days. All the iPS cell lines used in this study were characterized by alkaline phosphatase (AP) staining, karyotype analysis, pluripotency gene expression, cell differentiation ability and tetraploid complementation.

ES cells and iPS cells were cultured on mitomycin C-treated MEFs in ES medium, which contained (Dulbecco’s) modified Eagle’s medium ((D)MEM) (Gibco Invitrogen, Carlsbad, CA, USA) supplemented with 15% FBS, 1 mM L-glutamine, 0.1 mM mercaptoethanol, 1% non-essential amino acid stock, and 1,000 U/ml LIF (all from Chemicon, Temecula, CA, USA). Primary MEFs were obtained from 13.5-day embryos of ICR mice, based on the protocol from Wicell (Madison, WI, USA).

### Mice

All of our study procedures were consistent with the National Institute of Biological Sciences guide for the care and use of laboratory animals.

### RNA-seq

Total RNA was isolated from cell pellets using the TRIZOL reagent (Invitrogen, Grand Island, NY, USA) according to the manufacturer’s instructions. The RNA integrity was confirmed using a 2100 Bioanalyzer (Agilent Technologies, Santa Clara, CA, USA) with a minimum RNA integrity number (RIN) of 8. The mRNA was enriched using oligo(dT) magnetic beads and sheared to create short fragments of approximately 200 bp. cDNA was synthesized using random hexamer primers and purified using a PCR product extraction kit (Qiagen, Hilden, Germany). Finally, the cDNA fragments ligated with the sequencing primers (approximately 200 bp in total legth) were isolated by gel electrophoresis and enriched by PCR amplification to construct the library. The sequencing was performed at the Beijing Genomics Institute (BGI) (Shenzhen, Guangdong, China) using the HiSeqTM 2000 system developed by Illumina. For RNA-Seq, the cell lines were sequenced in two biological replicates to ensure that the results were highly reproducible. Paired-read sequencing was applied to RNA-Seq. The read counts are summarized in Additional file 8: Table S4.

### MNase-seq

Cells (1 × 10^7^) were suspended in 0.5 ml of TM buffer (10 mM Tris-HCl, 2 mM MgCl_2_, pH 7.5) and held on ice for 10 minutes. NP-40 was added to a final concentration of 1.5%, and the cells were incubated on ice for 10 minutes. The samples were then centrifuged at 2,000 rpm for four minutes to pellet the nuclei. The nuclei were washed once with TM buffer and resuspended in 200 μl of TM buffer, supplemented with CaCl_2_ to a final concentration of 1 mM. The nuclei were digested with 60 U of MNase at 37°C for 20 minutes. The digestion was halted by the addition of 2 mM ethylene glycol tetraacetic acid (EGTA), and the samples were incubated on ice for 10 minutes. The samples were then centrifuged at 2,000 rpm for four minutes to pellet the nuclei and were washed once with TM buffer. The nuclei pellet was resuspended in 0.5 ml STM600 buffer (10 mM Tris-HCl, pH 7.5, 2 mM EGTA, 2 mM MgCl_2_, 600 mM NaCl, 0.1% Triton-X100) and rotated at 4°C for two hours. The insoluble fraction was pelleted by centrifugation at 12,000 *g* for 10 minutes. The suspension was electrophoresed on a 2% agarose gel, and the bands containing mono-nucleosomes were excised and recovered using a Qiagen agarose gel recovery kit. The mono-nucleosomes were sequenced at BGI. The read counts are summarized in Additional file 9: Table S5.

### Gene expression analysis

The RNA-seq reads were mapped to the University of California at Santa Cruz (UCSC) genes (version mm9) using TopHat 2.0.4. All uniquely matching alignments were retained. Then, we used Cufflinks 2.0.2 to assemble the alignments into gene transcripts and to calculate their expression levels as reads per kilobase per million mapped reads (RPKM). For cell lines with two replicates, the average of the RPKM values from the two replicates was used. The RPKM values of the genes were used for cell-line pairwise correlation analysis of the gene expression profiles using a Spearman test.

### Nucleosome occupancy calculation

Sequence reads were mapped to the mouse reference genome (mm9) using Bowtie, and all uniquely matching reads were retained. The reads mapped to the sense strand and the reads mapped to the antisense strand were separated. Then, the RPKM at each site surrounding the TSSs was calculated and displayed in a heatmap. Additionally, a 10-kb window was used to scan all the chromosomes. The RPKM value of each window was calculated. In this calculation, reads mapped to both strands were used. The RPKM values of all the windows were compared pairwise between samples for the correlation analysis. Several offsets were used and returned similar results.

### Nucleosome distribution profiles

The gene annotation file was downloaded from the UCSC Genome Bioinformatics. The position of the nucleosome midpoint was defined as 73 bp downstream of the 5’ end of the read. Nucleosomes within 3,000 bp flanking the TSSs were collected. The distance of the nucleosome midpoint relative to the TSS was calculated and binned in 10-bp intervals. Bin data were normalized to the number of regions represented in each bin and the total number of retained uniquely matching reads; the data were then smoothed by five bins with a step size of one bin. Nucleosome distribution profiles surrounding the transcription factor binding sites were calculated in the same manner except that the center of the binding sites was used as the reference point.

### Accession numbers

The RNA-seq deep-sequencing data sets have been deposited in the National Center for Biotechnology Information’s Gene Expression Omnibus (GEO) under the accession number GSE46716. The raw sequence reads from the MNase-Seq analysis have been deposited in the Short Read Archive (SRA) under the accession number SRA075331.

## Additional files

Additional file 1: Figure S1. Generation of all-iPSC mice and high reproducibility of RNA-seq. A) Adult all-iPSC mice with germline transmission ability generated from the secondary iPSC lines S8 (left) and T2 (right). The other iPSC lines 16-6 and 32 also have developmental potentials to produce viable full-term all-iPSC mice (data not shown). B) High correlation of gene expression profiles of biological replicates. Additional file 2: Table S1. Generation of iPSC-tetraploid complemented pups and adult mice. Additional file 3: Figure S2. Similar chromatin landscape shared by mouse ESC and the secondary iPSCs. A) Heatmap for the difference in nucleosome occupancy around TSS by pairwise subtraction between two cell lines in Figure 2A. The darker less difference. Black indicates no difference. B) Nucleosome occupancy around TSS is highly reproducible in the biological replicates. C) Limited variations in nucleosome occupancy across the genome are shown in the representative comparisons (16-6 versus R1, and 32 versus 16-6). Conversely, there is significant difference in nucleosome occupancy between iPSC 16-6 and MEF. The genome is scanned with a 10-kb window. The ratio of normalized nucleosome occupancy within each window is presented by a color. Red indicates read count in cell line one is at least two-fold of that in cell line two. Green indicates read count in cell line two is at least two-fold of that in cell line one. Yellow indicates the ratio is less than two-fold. Grey indicates regions of Ns in the genome. D) Genes with RPKM = 0 are defined as silent genes. The rest are active genes. There is a -1, NDR, +1, +2, +3, and so on, canonical nucleosome arrangement around TSSs of active genes. In contrast, TSSs of silent genes are protected by a prominent nucleosome. Mouse iPS cell lines: 32, S8, T2. Additional file 4: Table S2. The correlation coefficients of global nucleosome occupancy between all cell lines. Additional file 5: Table S3. The correlation coefficients of nucleosome distribution around the ± 2 kb region of the TSSs. Additional file 6: Figure S3. Characteristic topological relationships between the transcription factor binding sites (TFBS) and nucleosome occupancy. There are four types of nucleosome occupancy patterns around the TFBS as defined in Figure 3. For simplicity, this figure only shows mouse iPS cell lines S8, T2 and their biological replicates with high reproducibility. Additional file 7: Figure S4. Nucleosome occupancy at classified loci. Nucleosome occupancy is similar to each other in the ESC R1 and iPSCs. Transcripts from RefSeq are shown under the tracks. Pluripotency transcription factors *Oct4*, *Sox2*, *Klf4*, *Myc*, *Nanog*, *Lin28a*; tissue-specific genes *Hoxa9*, *Igf1r*, *Krt5* and *Pgc*. Additional file 8: Table S4. The number of RNA-seq reads. Additional file 9: Table S5. The number of MNase-seq reads.
