# Supplementary material for: Nucleosome organizations in induced pluripotent stem cells reprogrammed from somatic cells belonging to three different germ layers
Source: BMC Biol. 2014 Dec 21;12:109. doi: 10.1186/s12915-014-0109-x (PMC4296552; doi:10.1186/s12915-014-0109-x)
Supplement: Additional file 1: Figure S1. — Generation of all-iPSC mice and high reproducibility of RNA-seq. A) Adult all-iPSC mice with germline transmission ability generated from the secondary iPSC lines S8 (left) and T2 (right). The other iPSC lines 16-6 and 32 also have developmental potentials to produce viable full-term all-iPSC mice (data not shown). B) High correlation of gene expression profiles of biological replicates. [file 12915_2014_109_MOESM1_ESM.doc]

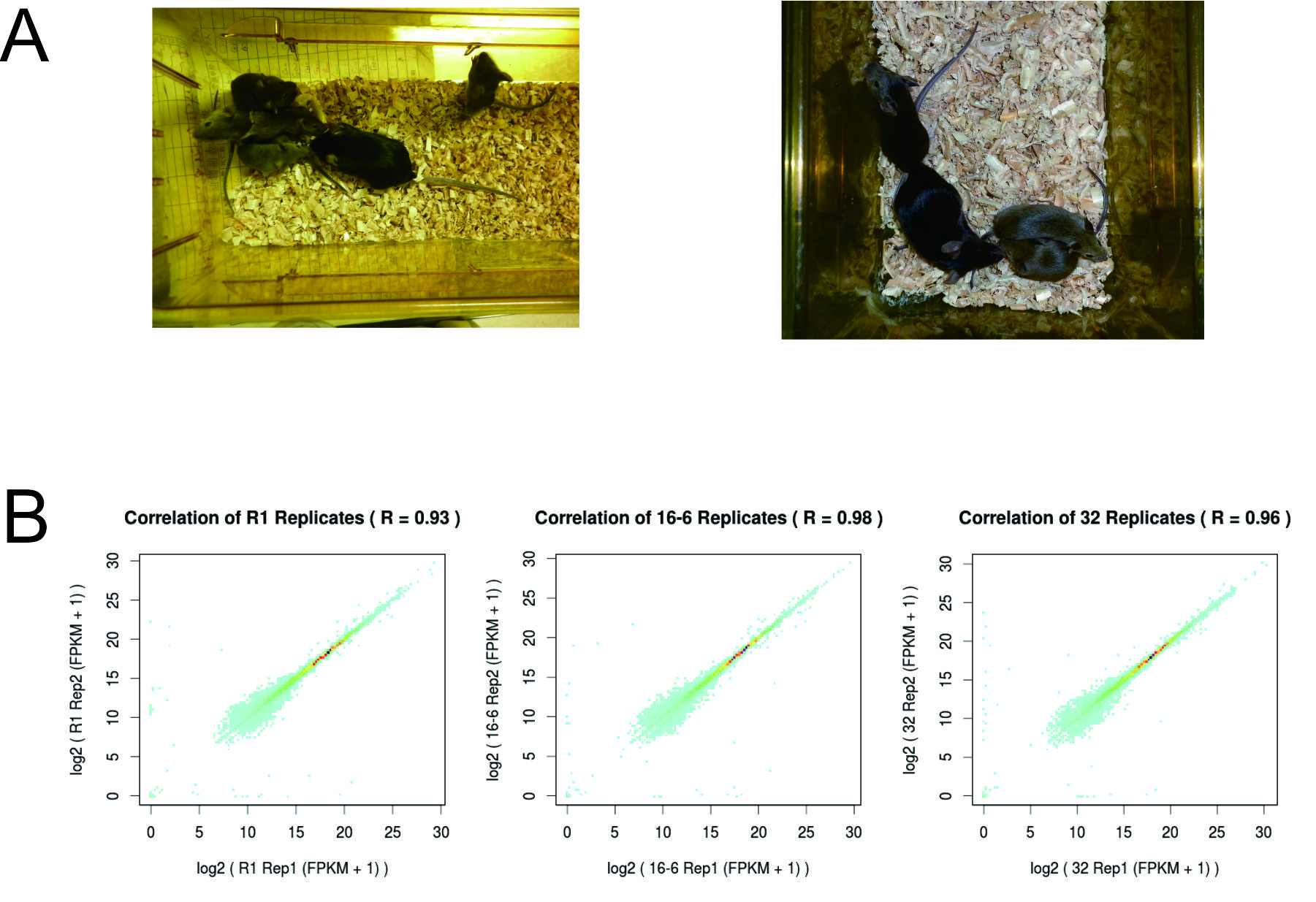


**Figure S1** **Generation of all-iPSC mice and high reproducibility of RNA-seq.**

(**A**) Adult all-iPSC mice with germline transmission ability generated from the secondary iPSC lines S8 (left) and T2 (right). The other iPSC lines 16-6 and 32 also have developmental potentials to produce viable full-term all-iPSC mice (data not shown). (**B**) High correlation of gene expression profiles of biological replicates.
