# Supplementary material for: Nucleosome organizations in induced pluripotent stem cells reprogrammed from somatic cells belonging to three different germ layers
Source: BMC Biol. 2014 Dec 21;12:109. doi: 10.1186/s12915-014-0109-x (PMC4296552; doi:10.1186/s12915-014-0109-x)
Supplement: Additional file 2: Table S1. — Generation of iPSC-tetraploid complemented pups and adult mice. [file 12915_2014_109_MOESM2_ESM.docx]

**Table S1** Generation of iPSC-tetraploid complemented pups and adult mice.

| tissue of parental fibroblast cells | 2° iPSC lines | No. 4N embryo implanted | No. pseudo-pregnant mother | No. E19.5 pups | No. E19.5 breathing pups | No. Adult mice |
| --- | --- | --- | --- | --- | --- | --- |
| Hematopoietic cells | 16-6 | 200 | 7 | 3 | 2 | 2 |
| Adipocyte progenitor cells | 32 | 755 | 25 | 14 | 10 | 2 |
| Epidermal cells | S8 | 251 | 17 | 12 | 8 | 6 |
| Stomach lining cells | T2 | 490 | 37 | 8 | 6 | 2 |
