# Supplementary material for: Nucleosome organizations in induced pluripotent stem cells reprogrammed from somatic cells belonging to three different germ layers
Source: BMC Biol. 2014 Dec 21;12:109. doi: 10.1186/s12915-014-0109-x (PMC4296552; doi:10.1186/s12915-014-0109-x)
Supplement: Additional file 3: Figure S2. — Similar chromatin landscape shared by mouse ESC and the secondary iPSCs. A) Heatmap for the difference in nucleosome occupancy around TSS by pairwise subtraction between two cell lines in Figure 2A. The darker less difference. Black indicates no difference. B) Nucleosome occupancy around TSS is highly reproducible in the biological replicates. C) Limited variations in nucleosome occupancy across the genome are shown in the representative comparisons (16-6 versus R1, and 32 versus 16-6). Conversely, there is significant difference in nucleosome occupancy between iPSC 16-6 and MEF. The genome is scanned with a 10-kb window. The ratio of normalized nucleosome occupancy within each window is presented by a color. Red indicates read count in cell line one is at least two-fold of that in cell line two. Green indicates read count in cell line two is at least two-fold of that in cell line one. Yellow indicates the ratio is less than two-fold. Grey indicates regions of Ns in the genome. D) Genes with RPKM = 0 are defined as silent genes. The rest are active genes. There is a -1, NDR, +1, +2, +3, and so on, canonical nucleosome arrangement around TSSs of active genes. In contrast, TSSs of silent genes are protected by a prominent nucleosome. Mouse iPS cell lines: 32, S8, T2. [file 12915_2014_109_MOESM3_ESM.doc]

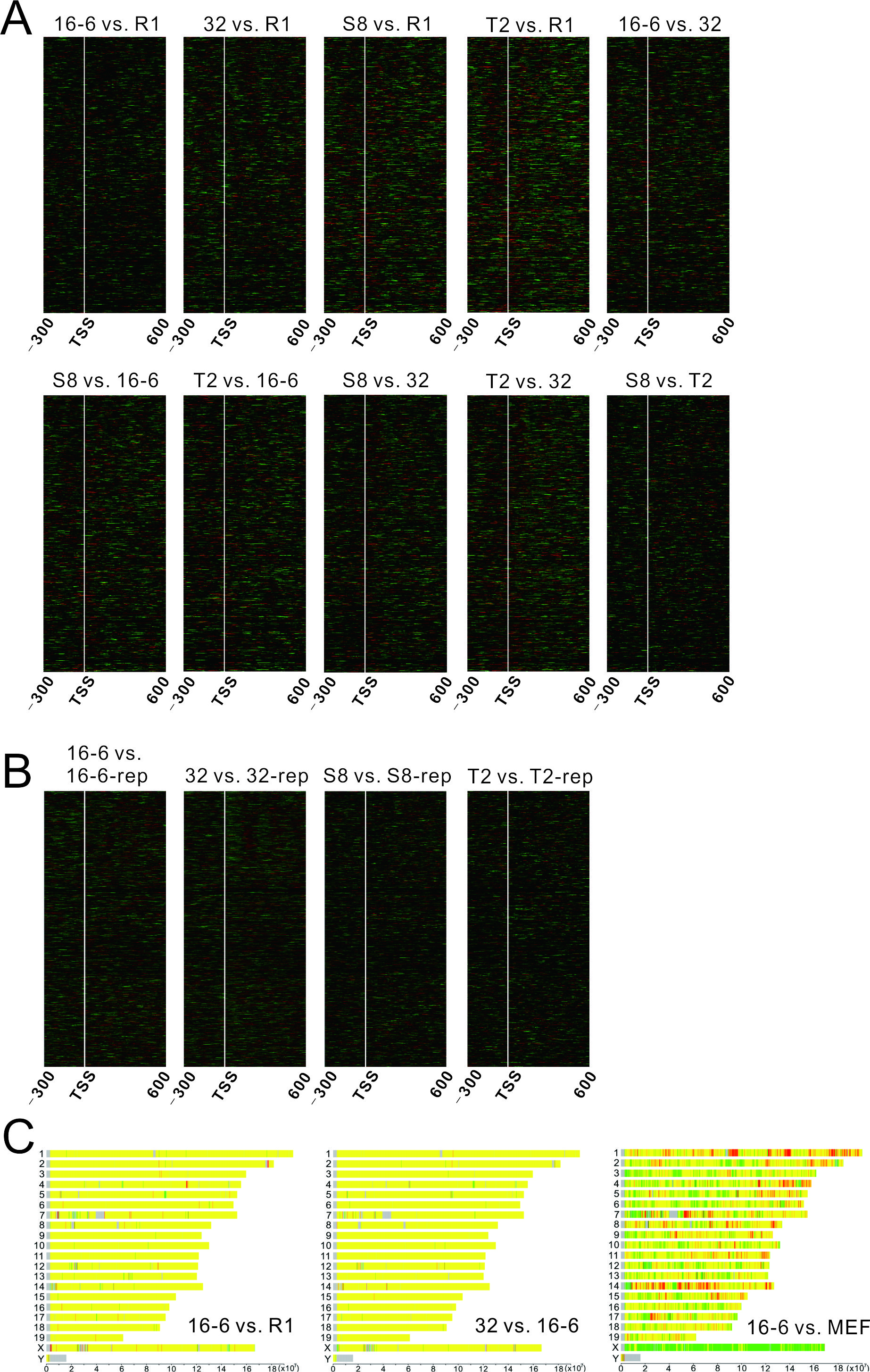

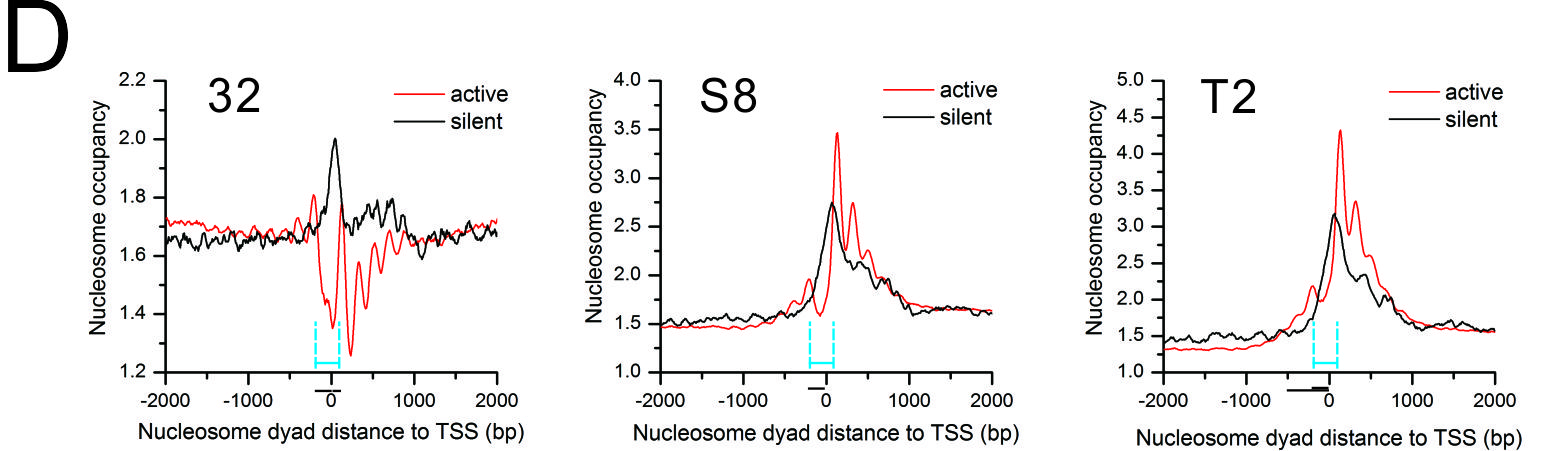


**Figure S2** **Similar chromatin landscape shared by mouse ESC and the secondary iPSCs.**

(**A**) Heatmap for the difference in nucleosome occupancy around TSS by pairwise subtraction between two cell lines in Figure 2A. The darker less difference. Black indicates no difference. (**B**) Nucleosome occupancy around TSS is highly reproducible in the biological replicates. (**C**) Limited variations in nucleosome occupancy across the genome are shown in the representative comparisons (16-6 VS. R1, and 32 VS. 16-6). Conversely, there is significant difference in nucleosome occupancy between iPSC 16-6 and MEF. The genome is scanned with a 10-kb window. The ratio of normalized nucleosome occupancy within each window is presented by a color. Red indicates read count in cell line one is at least 2 fold of that in cell line two. Green indicates read count in cell line two is at least 2 fold of that in cell line one. Yellow indicates the ratio is less than two fold. Grey indicates regions of Ns in the genome. (**D**) Genes with RPKM=0 are defined as silent genes. The rest are active genes. There is a -1, NDR, +1, +2, +3, etc, canonical nucleosome arrangement around TSSs of active genes. In contrast, TSSs of silent genes are protected by a well-positioned nucleosome. Mouse iPS cell lines: 32, S8, T2. The cyan lines indicate the region (-200bp ~ +100bp of TSS) with distinct nucleosome distribution patterns between active and silent genes that are conserved in all the pluripotent stem cell lines.
