# Supplementary material for: Nucleosome organizations in induced pluripotent stem cells reprogrammed from somatic cells belonging to three different germ layers
Source: BMC Biol. 2014 Dec 21;12:109. doi: 10.1186/s12915-014-0109-x (PMC4296552; doi:10.1186/s12915-014-0109-x)
Supplement: Additional file 4: Table S2. — The correlation coefficients of global nucleosome occupancy between all cell lines. [file 12915_2014_109_MOESM4_ESM.doc]

**Table S2** The correlation coefficient of global nucleosome occupancy between all cell lines (Spearman R-values).

| **Cell line** | **R1** | **16-6** | **16-6-rep** | **32** | **32-rep** | **S8** | **S8-rep** | **T2** | **T2-rep** | **ESC^a^** | **MEF^b^** |
| --- | --- | --- | --- | --- | --- | --- | --- | --- | --- | --- | --- |
| **R1** | 1 | 0.95196 | 0.93455 | 0.97919 | 0.95046 | 0.95167 | 0.95056 | 0.92533 | 0.92337 | 0.95288 | 0.43982 |
| **16-6** |  | 1 | 0.94659 | 0.95786 | 0.94672 | 0.94817 | 0.94732 | 0.92609 | 0.92439 | 0.94518 | 0.40632 |
| **16-6-rep** |  |  | 1 | 0.92712 | 0.99231 | 0.98676 | 0.98681 | 0.98791 | 0.98749 | 0.92481 | 0.32359 |
| **32** |  |  |  | 1 | 0.94508 | 0.94697 | 0.94563 | 0.91793 | 0.91584 | 0.95464 | 0.44433 |
| **32-rep** |  |  |  |  | 1 | 0.99339 | 0.99332 | 0.98921 | 0.98851 | 0.92665 | 0.34348 |
| **S8** |  |  |  |  |  | 1 | 0.99802 | 0.99243 | 0.99178 | 0.91411 | 0.33999 |
| **S8-rep** |  |  |  |  |  |  | 1 | 0.99277 | 0.99217 | 0.91271 | 0.33891 |
| **T2** |  |  |  |  |  |  |  | 1 | 0.99830 | 0.87510 | 0.30255 |
| **T2-rep** |  |  |  |  |  |  |  |  | 1 | 0.87308 | 0.30078 |

^a^ The nucleosome data of ESCs is from the published literature .

^b^ The MEF nucleosome data is from the published study .
