# Supplementary material for: Nucleosome organizations in induced pluripotent stem cells reprogrammed from somatic cells belonging to three different germ layers
Source: BMC Biol. 2014 Dec 21;12:109. doi: 10.1186/s12915-014-0109-x (PMC4296552; doi:10.1186/s12915-014-0109-x)
Supplement: Additional file 5: Table S3. — The correlation coefficients of nucleosome distribution around the ± 2 kb region of the TSSs. [file 12915_2014_109_MOESM5_ESM.doc]

**Table S3A** Correlation of nucleosome distribution around the ±2kb region of the TSSs of active genes (Pearson R-values)

|  | R1 | 16-6 | 32 | S8 | T2 |
| --- | --- | --- | --- | --- | --- |
| R1 | 1 | 0.829231 | 0.840341 | 0.662468 | 0.5338 |
| 16-6 |  | 1 | 0.829135 | 0.769773 | 0.645954 |
| 32 |  |  | 1 | 0.620409 | 0.500643 |
| S8 |  |  |  | 1 | 0.942306 |
| T2 |  |  |  |  | 1 |

**Table S3B** Correlation of nucleosome distribution around the ±2kb region of the TSSs of unexpressed genes (Pearson R-values)

|  | R1 | 16-6 | 32 | S8 | T2 |
| --- | --- | --- | --- | --- | --- |
| R1 | 1 | 0.873723 | 0.897582 | 0.80761 | 0.656701 |
| 16-6 |  | 1 | 0.89946 | 0.882186 | 0.76232 |
| 32 |  |  | 1 | 0.834813 | 0.67741 |
| S8 |  |  |  | 1 | 0.894292 |
| T2 |  |  |  |  | 1 |
