# Supplementary material for: Nucleosome organizations in induced pluripotent stem cells reprogrammed from somatic cells belonging to three different germ layers
Source: BMC Biol. 2014 Dec 21;12:109. doi: 10.1186/s12915-014-0109-x (PMC4296552; doi:10.1186/s12915-014-0109-x)
Supplement: Additional file 6: Figure S3. — Characteristic topological relationships between the transcription factor binding sites (TFBS) and nucleosome occupancy. There are four types of nucleosome occupancy patterns around the TFBS as defined in Figure 3. For simplicity, this figure only shows mouse iPS cell lines S8, T2 and their biological replicates with high reproducibility. [file 12915_2014_109_MOESM6_ESM.doc]

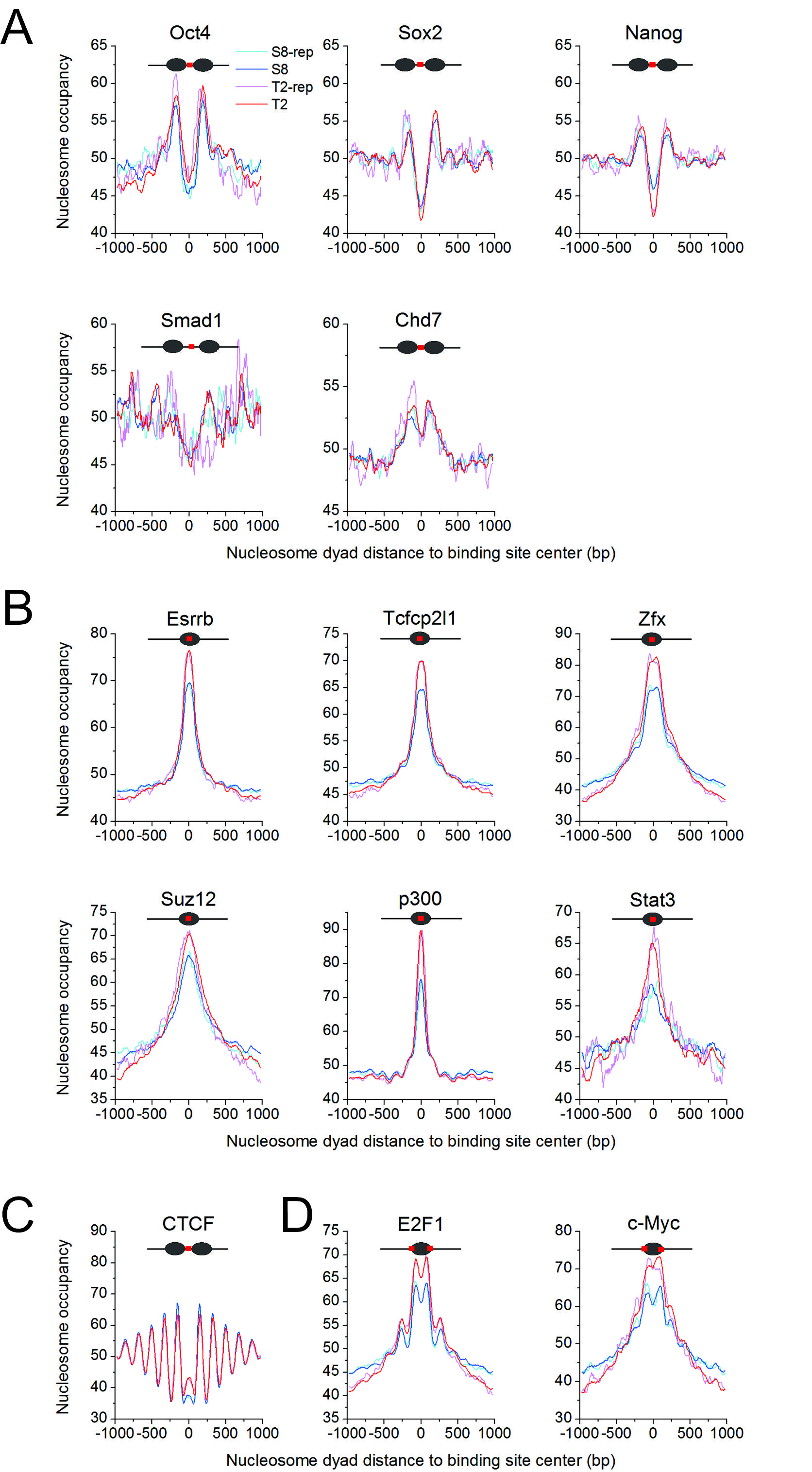


**Figure S3** **Characteristic topological relationships between the transcription factor binding sites (TFBS) and nucleosome organization.**

There are four types of nucleosome occupancy patterns around the TFBS as defined in Figure 3 (**ABCD**). For simplicity, here only shows mouse iPS cell lines S8, T2, and their biological replicates with high reproducibility.
