# Supplementary material for: Nucleosome organizations in induced pluripotent stem cells reprogrammed from somatic cells belonging to three different germ layers
Source: BMC Biol. 2014 Dec 21;12:109. doi: 10.1186/s12915-014-0109-x (PMC4296552; doi:10.1186/s12915-014-0109-x)
Supplement: Additional file 7: Figure S4. — Nucleosome occupancy at classified loci. Nucleosome occupancy is similar to each other in the ESC R1 and iPSCs. Transcripts from RefSeq are shown under the tracks. Pluripotency transcription factors Oct4, Sox2, Klf4, Myc, Nanog, Lin28a; tissue-specific genes Hoxa9, Igf1r, Krt5 and Pgc. [file 12915_2014_109_MOESM7_ESM.doc]

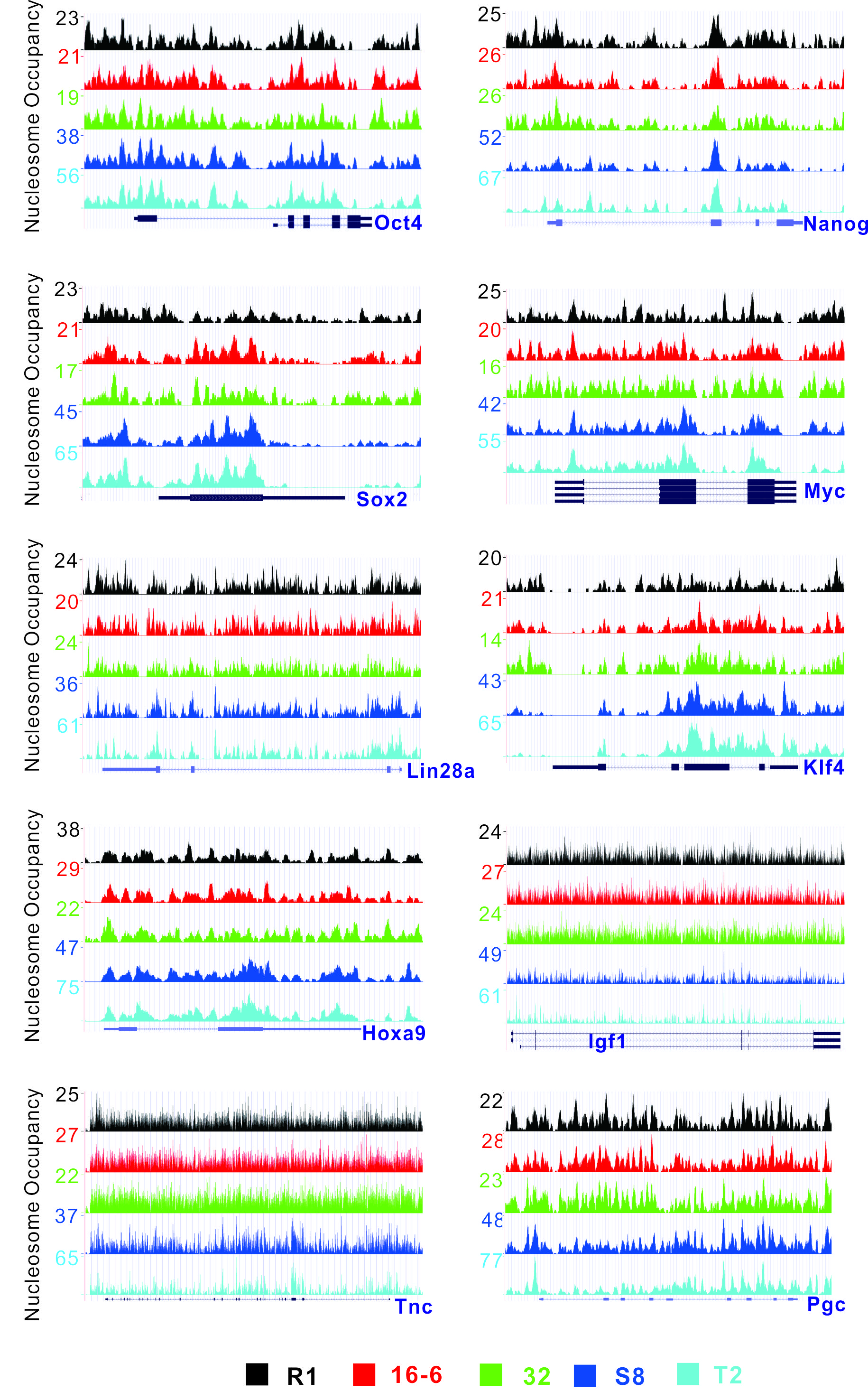


**Figure S4** **Nucleosome occupancy at classified loci.**

Nucleosome occupancy is similar to each other in the ESC R1 and iPSCs. Transcripts from RefSeq are shown under the tracks. Pluripotency transcription factors Oct4, Sox2, Klf4, Myc, Nanog, Lin28a; tissue-specific genes Hoxa9, Igf1r, Krt5, and Pgc.
