# Supplementary material for: Nucleosome organizations in induced pluripotent stem cells reprogrammed from somatic cells belonging to three different germ layers
Source: BMC Biol. 2014 Dec 21;12:109. doi: 10.1186/s12915-014-0109-x (PMC4296552; doi:10.1186/s12915-014-0109-x)
Supplement: Additional file 8: Table S4. — The number of RNA-seq reads. [file 12915_2014_109_MOESM8_ESM.doc]

**Table S4** The number of RNA-seq reads.

| Cell line | Replicate | Total Read | Uniquely Mapped Read | Percentage of Uniquely Mapped (%) |
| --- | --- | --- | --- | --- |
| R1 | rep1 | 24,190,976 | 20,578,373 | 85.07 |
| R1 | rep2 | 25,177,057 | 21,504,776 | 85.41 |
| 16-6 | rep1 | 23,918,551 | 20,627,307 | 86.24 |
| 16-6 | rep2 | 23,840,419 | 20,651,569 | 86.62 |
| 32 | rep1 | 24,198,252 | 20,452,803 | 84.52 |
| 32 | rep2 | 24,660,561 | 20,949,728 | 84.95 |
| S8 | rep1 | 91,722,592 | 64,484,659 | 70.30 |
| T2 | rep1 | 97,658,608 | 69,721,299 | 71.39 |

Note: ≤2 mismatches are allowed.
