# Supplementary material for: Nucleosome organizations in induced pluripotent stem cells reprogrammed from somatic cells belonging to three different germ layers
Source: BMC Biol. 2014 Dec 21;12:109. doi: 10.1186/s12915-014-0109-x (PMC4296552; doi:10.1186/s12915-014-0109-x)
Supplement: Additional file 9: Table S5. — The number of MNase-seq reads. [file 12915_2014_109_MOESM9_ESM.doc]

**Table S5** The number of MNase-seq reads.

| Cell line | Total Read | Uniquely Mapped Reads | Percentage of Uniquely Mapped (%) |
| --- | --- | --- | --- |
| R1 | 271,251,960 | 203,212,347 | 74.92 |
| 16-6 | 334,334,498 | 251,304,249 | 75.17 |
| 32 | 334,106,071 | 244,703,163 | 73.24 |
| S8 | 331,310,014 | 248,404,203 | 74.98 |
| T2 | 313,928,278 | 228,693,379 | 72.85 |
| 16-6-rep | 333,502,298 | 205,575,558 | 61.64 |
| 32-rep | 396,253,357 | 256,097,439 | 64.63 |
| S8-rep | 323,493,550 | 242,569,243 | 74.98 |
| T2-rep | 331,629,719 | 242,133,802 | 73.01 |

Note: ≤2 mismatches are allowed.
